# Evaluation of pathogenicity of *WT1* intron variants by in vitro splicing analysis

**DOI:** 10.1007/s10157-024-02510-w

**Published:** 2024-06-14

**Authors:** Seiya Inoue, Atsushi Kondo, Yuta Inoki, Yuta Ichikawa, Yu Tanaka, Chika Ueda, Hideaki Kitakado, Ryota Suzuki, Eri Okada, Nana Sakakibara, Tomoko Horinouchi, Kandai Nozu

**Affiliations:** 1 Department of Pediatrics, Kobe University Graduate School of Medicine, 7-5-1 Kusunoki-Cho, Chuo, Kobe, Hyogo 650-0017 Japan; 2 Department of Pediatrics, Hokkaido University Graduate School of Medicine, Sapporo, Japan; 3 Department of Nephrology, Faculty of Medicine, University of Tsukuba, Tsukuba, Japan

**Keywords:** *WT1* gene, Intronic variant, Splicing aberration, Pathogenicity, Minigene assay

## Abstract

**Background:**

Wilms tumor 1 (*WT1*; NM_024426) causes Denys–Drash syndrome, Frasier syndrome, or isolated focal segmental glomerulosclerosis. Several *WT1* intron variants are pathogenic; however, the pathogenicity of some variants remains undefined. Whether a candidate variant detected in a patient is pathogenic is very important for determining the therapeutic options for the patient.

**Methods:**

In this study, we evaluated the pathogenicity of *WT1* gene intron variants with undetermined pathogenicity by comparing their splicing patterns with those of the wild-type using an in vitro splicing assay using minigenes. The three variants registered as likely disease-causing genes: Mut1 (c.1017-9 T > C(IVS5)), Mut2 (c.1355-28C > T(IVS8)), Mut3 (c.1447 + 1G > C(IVS9)), were included as subjects along the 34 splicing variants registered in the Human Gene Mutation Database (HGMD)^®^.

**Results:**

The results showed no significant differences in splicing patterns between Mut1 or Mut2 and the wild-type; however, significant differences were observed in Mut3.

**Conclusion:**

We concluded that Mut1 and Mut2 do not possess pathogenicity although they were registered as likely pathogenic, whereas Mut3 exhibits pathogenicity. Our results suggest that the pathogenicity of intronic variants detected in patients should be carefully evaluated.

**Supplementary Information:**

The online version contains supplementary material available at 10.1007/s10157-024-02510-w.

## Introduction

Recent advances in genetic analyses have revealed that some intron variants become pathogenic via aberrant mRNA splicing [1–3]. An in vivo transcript expression analysis using specimens from affected patients is the most reliable approach for detecting splicing aberrations. Although transcript expression analysis, such as reverse transcription polymerase chain reaction (RT-PCR), is necessary for determining the pathogenicity of novel intronic variants, obtaining appropriate specimens for RT-PCR is usually extremely difficult for genes with low mRNA expression in peripheral lymphocytes, such as Wilms tumor 1 (*WT1*).

*WT1* (NM_024426) is located on chromosome 11p13 and has an autosomal dominant pattern of inheritance. It encodes a zinc finger transcription factor involved in kidney and gonadal development during early embryonic stages [4]. Moreover, pathogenic *WT1* gene variants cause kidney diseases, such as diffuse mesangial sclerosis and focal segmental glomerulosclerosis (FSGS), which can lead to end-stage kidney disease (ESKD). In addition, it can cause external genitalia, Wilms tumor, and gonadoblastoma, Denis–drash syndrome (DDS), and frasier syndrome (FS) [5–7]. FS, a rare disorder caused by variants of the donor splice site located in intron 9 of *WT1*, is characterized by kidney diseases such as steroid-resistant nephrotic syndrome (SRNS), external genitalia, and gonadoblastoma [8, 9]. One of the alternative splicing patterns of *WT1* is the presence or absence of three amino acids between zinc finger 3 and zinc finger 4 (+ KTS or − KTS) governed by a 5’ splice junction [10]. Normally, the ratio of + KTS and − KTS is 1:0.85. However, the ratio deviates to 1:3 in patients with FS, and the FS-associated mutational alleles exclusively yield the − KTS transcript [11]. Although several studies have reported *WT1* gene intron variants, the pathogenicity of all intron variants has not been defined yet.

In this study, we determined the pathogenicity of intronic variants with undefined pathogenicity and already registered in the database using in vitro splicing assays using minigenes.

## Materials and methods

**Fig. 1:**
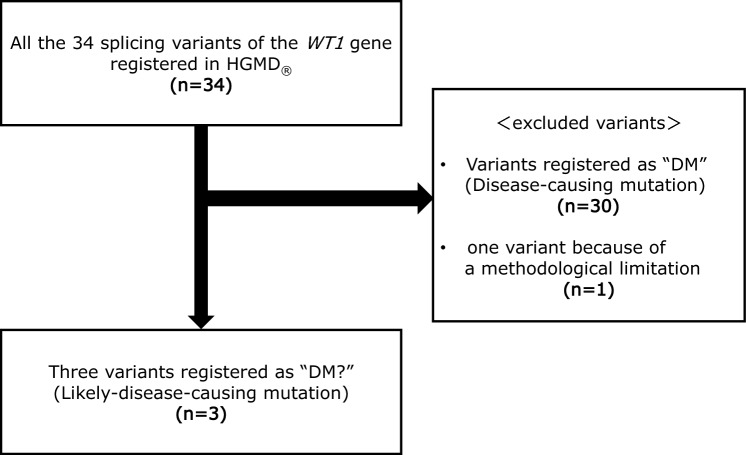
Method of selection of the subject of this study

Thirty-four splicing variants have been registered in the Human Gene Mutation Database (HGMD^®^, http://www.hgmd.org) professional (2023.3), which is a genome database available on the internet. Among them, we selected only variants that HGMD has marked as “DM?” due to doubts regarding their pathogenicity. There are 4 variants labeled as “DM?” in the registry (Supplementary Table 1). However, variants located in introns flanking the first or last exon are challenging to assess using the minigene assay because they are not bounded by canonical splice site sequences (AG–GT). The minigene assay can only accurately assess exons flanked by AG–GT splice sites. Consequently, the variant c.1448-10G > A, affecting the terminal exon (Exon 10), was excluded from this study. Therefore, the remaining three mutants included in this study were Mut1 (c.1017-9 T > C, IVS5), Mut2 (c.1355-28C > T, IVS8), and Mut3 (c.1447 + 1G > C, IVS9; Fig. 1). Although Mut1 is registered as causing ‘hypospadias’ but not nephritis, it is noteworthy that ‘hypospadias’ is also recognized as a symptom associated with *WT1* variants [12]. Therefore, we included this variant in our study. In silico analysis for these variants were performed using MaxEntScan (MES) [13] (http://hollywood.mit.edu/burgelab/maxent/Xmaxentscan_scoreseq.html), SpliceSiteFinder-like (SSF; http://www.interactive-biosoftware.com), NNSPLICE [14] with Alamut Visual Plus (http://www.interactive-biosoftware.com/alamut-visual/), and splice AI (https://spliceailookup.broadinstitute.org/) [15]. We set > 10% for MES, > 5% for SSF and NNSPLICE, and 0.2 for splice AI as the cutoff values [16–18].

Hybrid minigene constructs were generated using the H492v vector, a tool developed in our previous study [19]. First, wild-type gDNA fragments surrounding the target variants in the *WT1* gene were cloned by In-Fusion cloning methods with In-Fusion® HD Cloning Kit (Takara Bio Inc). Subsequently, site-directed mutagenesis was induced in the variants using the PrimeSTAR mutagenesis basal kit (Takara Bio Inc.) and primer sets (Supplementary Table 2).

The created plasmid vectors, Wild-type1, Wild-type2, and Mut1 to Mut3, were transfected into HEK293T cells by Lipofectamine® 3000 (Thermo Fisher Scientific, Waltham, MA, USA). After 24 h of culture, total RNA transcribed by the HEK293T cells was extracted with RNeasy® Plus Mini Kit (250) (QIAGEN, GmbH, Hilden, Germany). Total RNA (1 μg pr sample) was reverse-transcribed using the RNA to cDNA EcoDry™ Premix (Double Primed) kit (Takara Bio Inc). The product lengths were confirmed using agarose gel electrophoresis with φX174-Hae III digest marker (Takara Bio Inc.) and their sequence was analyzed by Sanger sequencing.

In addition, the combinations with no obvious difference in splicing patterns between Wild-type and Mut type based on electrophoresis and Sanger sequencing were examined using Agilent2100 Bioanalyzer (Biosizing software, Ver. A.01.05) with Agilent High Sensitivity DNA kit to evaluate the significant differences in splicing patterns, including the − KTS to + KTS.

## Results

The results of in silico analyses predicted that Mut1 and Mut2 did not cause aberrant splicing, and only Mut3 caused aberrant splicing (Table 1). In addition, for the variant excluded from our study as described previously, c.1448-10G > A, to further elucidate its pathogenicity, we conducted in silico analysis, and the results are added to Supplementary Table 1. Based on these findings of in silico analyses, it was determined to be not likely pathogenic.

**Table 1:** Summary of subject variants: results of in silico analysis and minigene assay

No	Variant (IVS)	Prediction by In silico analyses							Final classifications by minigene assay	Confliction between in silico analyses and minigene assay results (y/n)
		MES (%)	SSF-like (%)	NNSPLICE (%)	Splice AI scores(0–1)					
					Acceptor loss	Donor loss	Acceptor gain	Donor gain		
Mut1	c.1017-9 T > C (IVS5)	+ 4.0 (Acceptor site)	− 3.0 (Acceptor site)	− 3.0 (Acceptor site)	0	0	0	0	Normal splicing pattern	n
Mut2	c.1355-28C > T (IVS8)	0	0	0	0	0	0	0.11	Normal splicing pattern	n
Mut3	c.1447 + 1G > C (IVS9)	-100 (Donor site)	− 100 (Donor site)	− 100 (Donor site)	0	0.83	0.01	0.02	Aberrant splicing pattern	n

*IVS* intervening sequence

**Fig. 2:**
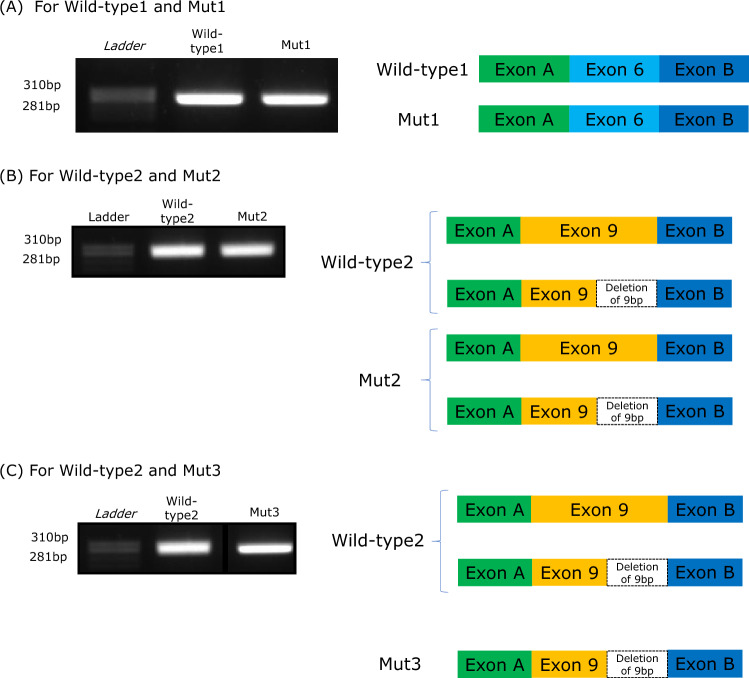
The results of electrophoresis and schematic transcript analysis of minigene assay products. **A** For Wild-type1 and Mut1, **B** For Wild-type2 and Mut2, **C** For Wild-type2 and Mut3.

The splicing pattern for Wild-type1 and Mut1 was not different and both transcript products exhibited the wild-type splicing pattern (Fig. 2 (A)). Moreover, Wild-type2 and Mut2 transcript products showed identical sequences and expressed + KTS and -KTS patterns (Fig. 2 (B)). Furthermore, additional examination using a bioanalyzer showed that the + KTS to − KTS ratio in Wild-type2 and Mut2 was 1:0.82 and 1:0.71, respectively. Therefore, the splicing pattern of Wild-type2 and Mut2 was not clearly distinct. Interestingly, the Wild-type2 transcript products included + KTS and − KTS patterns, whereas Mut3 expressed only the − KTS pattern (Fig. 2 (C)).

Based on these results, we conclude that only Mut3 is pathogenic.

## Discussion

In this study, the pathogenicity of three intron variants with undetermined pathogenicity in HGMD was evaluated using a minigene assay; two variants with no difference in splicing pattern from the wild type were determined to be nonpathogenic, and only one variant with a clear difference was determined to be pathogenic. In addition, to examine the validity of the present results, we referred to articles that reported each variant to confirm their phenotypes (Table 2).

**Table 2:** Clinical manifestations of subject variants in previous reported cases

No	Previous report	Patient ID	Age at onset	Sex (M/F)	Phenotype of the nephrological and urological system	Pathological findings of kidney	Kidney disfunction
Mut1	Diposarosa R, et al. *J Pediatr Urol*. 2018 Jun;14(3):237	5	N/D*	M	mid-shaft hypospadias	N/D	N/D
		6	N/D	M	mid-shaft hypospadias	N/D	N/D
		41	N/D	M	penoscrotal hypospadias	N/D	N/D
Mut2	Bezdíčka M, et al. *Pediatr Nephrol*. 2018 Aug;33(8):1347–1363	2016	16	M	SRNS	FSGS	+ (CKD stage2)
Mut3	Gast C, et al. *Nephrol Dial Transplant*. 2016 Jun;31(6):961–70	I12	N/D	F	SRNS	FSGS	+ (ESKD at the age of 16)

*N/D no data

Mut1 has been detected in three patients [20]. In this study, all exons and introns surrounding exons in the *WT1* gene of 74 patients with hypospadias at a single facility were analyzed using Sanger sequencing. We performed only in silico analysis to evaluate the pathogenicity of the detected variants. Two of the three patients with Mut1 presented only with hypospadias, whereas the other had hypospadias, undescended testes, and anorectal malformations; however, other details were not described. Since only the *WT1* gene sequence was analyzed in this study, other unexamined genes may be involved in the phenotypes of these patients. Hypospadias is the second most common male congenital malformation after undescended testes, but the cause is not clear in most individuals, and hormonal influences as well as a single gene are suspected [21]. Moreover, in silico analysis, which was the basis for pathogenicity determination, was also considered Mut1 to be nonpathogenic in our investigation (Table 1). Based on these results, we conclude that a previous report on Mut1 does not contradict the results of our study.

Mut2 has been detected in only one patient [22]. In this study, targeted next-generation sequencing (NGS) and Sanger sequencing were performed on a patient with SRNS from multiple facilities. Mut2 was detected in a 16-year-old male with SRNS refractory to immunosuppressive therapy with cyclosporin A. He showed no Wilms’ tumor or other extrarenal manifestations, and no genitourinary abnormalities characteristic of FS. For variants that change + KTS and − KTS splicing patterns, the phenotype is expected to be FS owing to the abnormal ratio [10, 11]. However, in a previous study, Mut2 did not exhibit an FS-like phenotype [22]. Most importantly, no abnormalities in this ratio were observed using semi-quantitative PCR. As mentioned above, the transcript pattern of Mut2 in our in vitro assay expressed + KTS and − KTS, which was almost the same as that of the wild type. Based on these findings, we considered this to be a nonpathogenic variant.

Mut3 has also been detected in only one patient [23]. In this study, targeted NGS and Sanger sequencing of 39 genes were performed in 81 patients with FSGS and/or SRNS registered at a single facility. The patient with the Mut3 variant was a 16-year-old female with SRNS, and her pathological finding was FSGS. She had already developed ESKD at that time. Moreover, in several reports of a single nucleotide variant at the same position, c.1447 + 1G > A, additional analyses including minigene assay have been performed and confirmed to be pathogenic. This variant caused the same aberrant splicing (− KTS pattern) with Mut3. Although detailed information about the patient, such as her karyotype and extrarenal symptoms, including genitourinary abnormalities, is unknown, the pathological findings and her poor renal prognosis do not conflict with our finding that Mut3 is a pathogenic variant.

Therefore, we concluded that none of the reported phenotypes of Mut1/2/3 conflicted with the results of in vitro assay.

Determining the pathogenicity of novel and previously reported variants is crucial; however, their pathogenicity has not been determined for two main reasons. First, it is helpful in determining therapeutic strategies. Immunosuppressive therapy is generally recommended as a treatment for SRNS, but often ineffective for SRNS caused by single-gene pathogenic variants such as *WT1* [24, 25]. In rare cases, specific therapies may exist for mitochondrial diseases caused by single-gene pathogenic variants such as *COQ2*, *COQ6*, or *COQ8b* [26]. In addition, this information is important when selecting a donor for renal transplantation, considering the possibility of post-transplant recurrence. Moreover, genetic diagnosis is useful for predicting kidney prognosis and detecting extrarenal complications properly and earlier such as gonadoblastoma and Wilms tumor in patients with *WT1* gene variants [27–29]. Second, it provides critical information regarding inheritance in the next generation. In particular, there is a 50% likelihood that the genes with an autosomal dominant mode of inheritance, such as *WT1*, will be carried to the offspring. Therefore, this must be carefully considered when providing genetic counseling. The minigene assay is a relatively simple method to confirm whether an intron variant has a splicing abnormality, but it is not clear whether the same splice result actually occurs in the target tissue of a patient without mRNA analysis using target tissues. On the other hand, the accuracy of the minigene assay has been reported for many genes, and we consider it to be useful. In cases where variants present challenges for assessment via the minigene assay, it is preferable to validate splicing abnormalities through mRNA analysis using patient peripheral blood or renal tissue. However, obtaining such specimens can pose logistical challenges. Segregation analysis also offers valuable insights; nevertheless, assembling cases with rare variants for this purpose can be daunting. An alternative strategy involves conducting in silico analysis. The finding in this study is consistent with the results of in silico analyses, thereby confirming their utility.

Our study had some limitations. First, the minigene assay is not appropriate for assessing the first or last exons of genes because the splicing acceptor or donor site of the first or last exon differs from that of the other exons. Thus, we excluded one intronic variant that existed around the last exon. Second, the minigene assay is limited by the length of DNA fragments that can be inserted into the vector. Therefore, in this study, only the exon closest to the target variant was inserted. The results might have been different if we had inserted a few exons. Third, whether the same splicing occurs in vivo or in vitro is unclear. Ideally, the results of the in vitro analysis should also be confirmed by in vivo analysis, such as mRNA analysis using urinary drop cells or renal biopsy specimens. From our previous results, most of the minigene assay results were consistent with the mRNA sequence of patient tissues [19, 30, 31].

However, since obtaining such specimens is often difficult, performing a minigene assay, as in this study, would be useful to determine whether the results are consistent with the results of in silico analysis or previously reported phenotypes.

## Conclusion

We used a minigene assay to evaluate three *WT1* intronic variants suspected to be pathogenic and determined that only one of them was pathogenic. The pathogenicity of an intron variant detected in a database or a patient is clinically important and must be carefully determined.

Ideally, in vivo analysis using patient specimens, such as renal tissues associated with the symptoms, would be desirable; however, because specimens are often difficult to obtain, an in vitro analysis such as that used in this study is useful.

## Supplementary Information

Below is the link to the electronic supplementary material.

Supplementary file1 (PPTX 8810 KB)
