# Supplementary material for: Evaluation of pathogenicity of WT1 intron variants by in vitro splicing analysis
Source: Clin Exp Nephrol. 2024 Jun 14;28(11):1075–81. doi: 10.1007/s10157-024-02510-w (PMC11568005; doi:10.1007/s10157-024-02510-w)
Supplement: Supplementary file 1 — Supplementary file1 (PPTX 8810 KB) [file 10157_2024_2510_MOESM1_ESM.pptx]

## Slide 1
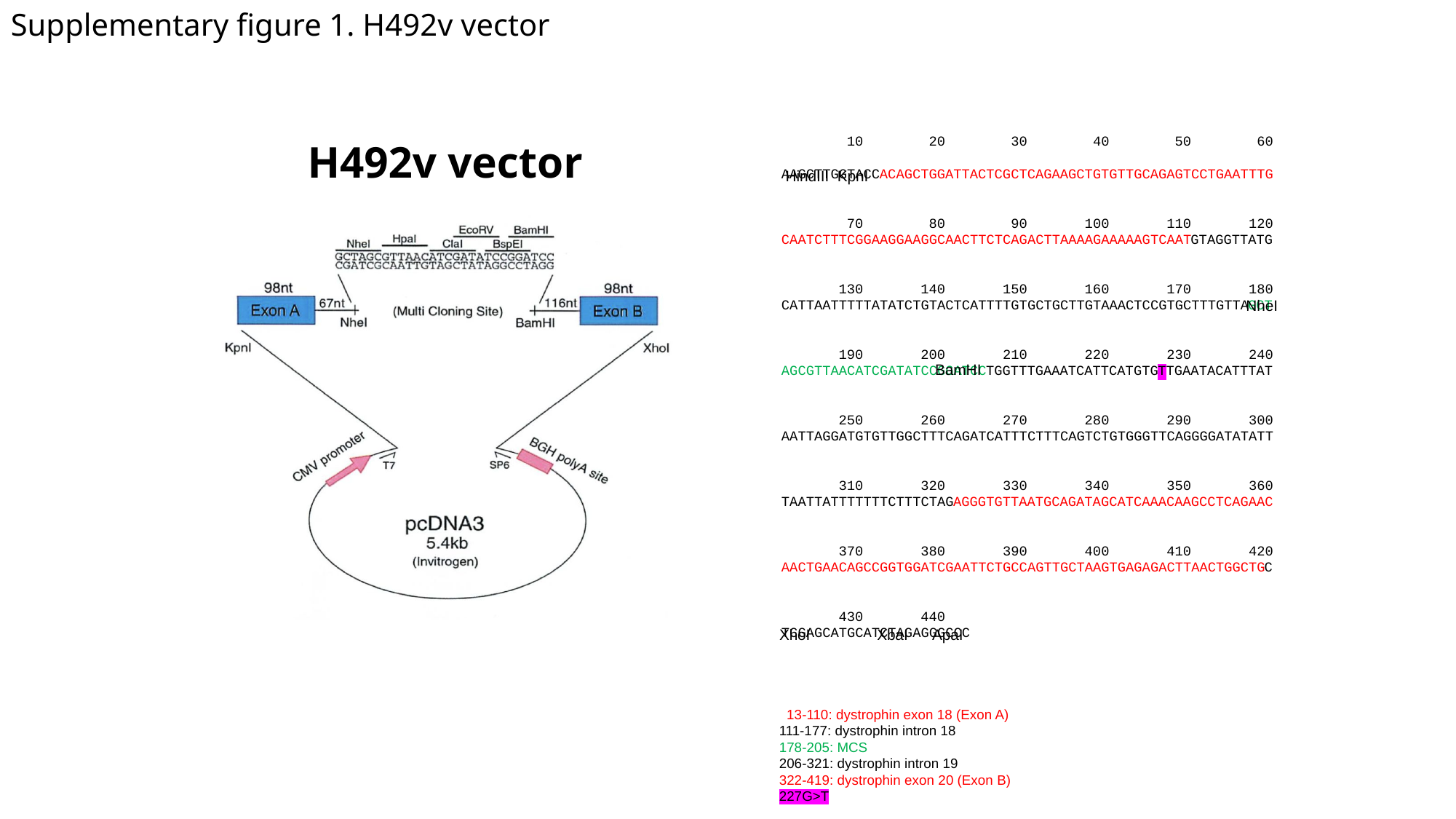

Supplementary figure 1. H492v vector
 10 20 30 40 50 60
AAGCTTGGTACCACAGCTGGATTACTCGCTCAGAAGCTGTGTTGCAGAGTCCTGAATTTG
 70 80 90 100 110 120
CAATCTTTCGGAAGGAAGGCAACTTCTCAGACTTAAAAGAAAAAGTCAATGTAGGTTATG
 130 140 150 160 170 180
CATTAATTTTTATATCTGTACTCATTTTGTGCTGCTTGTAAACTCCGTGCTTTGTTAGCT
 190 200 210 220 230 240
AGCGTTAACATCGATATCCGGATCCTGGTTTGAAATCATTCATGTGTTGAATACATTTAT
 250 260 270 280 290 300
AATTAGGATGTGTTGGCTTTCAGATCATTTCTTTCAGTCTGTGGGTTCAGGGGATATATT
 310 320 330 340 350 360
TAATTATTTTTTTCTTTCTAGAGGGTGTTAATGCAGATAGCATCAAACAAGCCTCAGAAC
 370 380 390 400 410 420
AACTGAACAGCCGGTGGATCGAATTCTGCCAGTTGCTAAGTGAGAGACTTAACTGGCTGC
 430 440
TCGAGCATGCATCTAGAGGGCCC
HindIII KpnI
NheI
BamHI
XhoI XbaI ApaI
H492v vector
 13-110: dystrophin exon 18 (Exon A)
111-177: dystrophin intron 18
178-205: MCS
206-321: dystrophin intron 19
322-419: dystrophin exon 20 (Exon B)
227G>T

## Slide 2
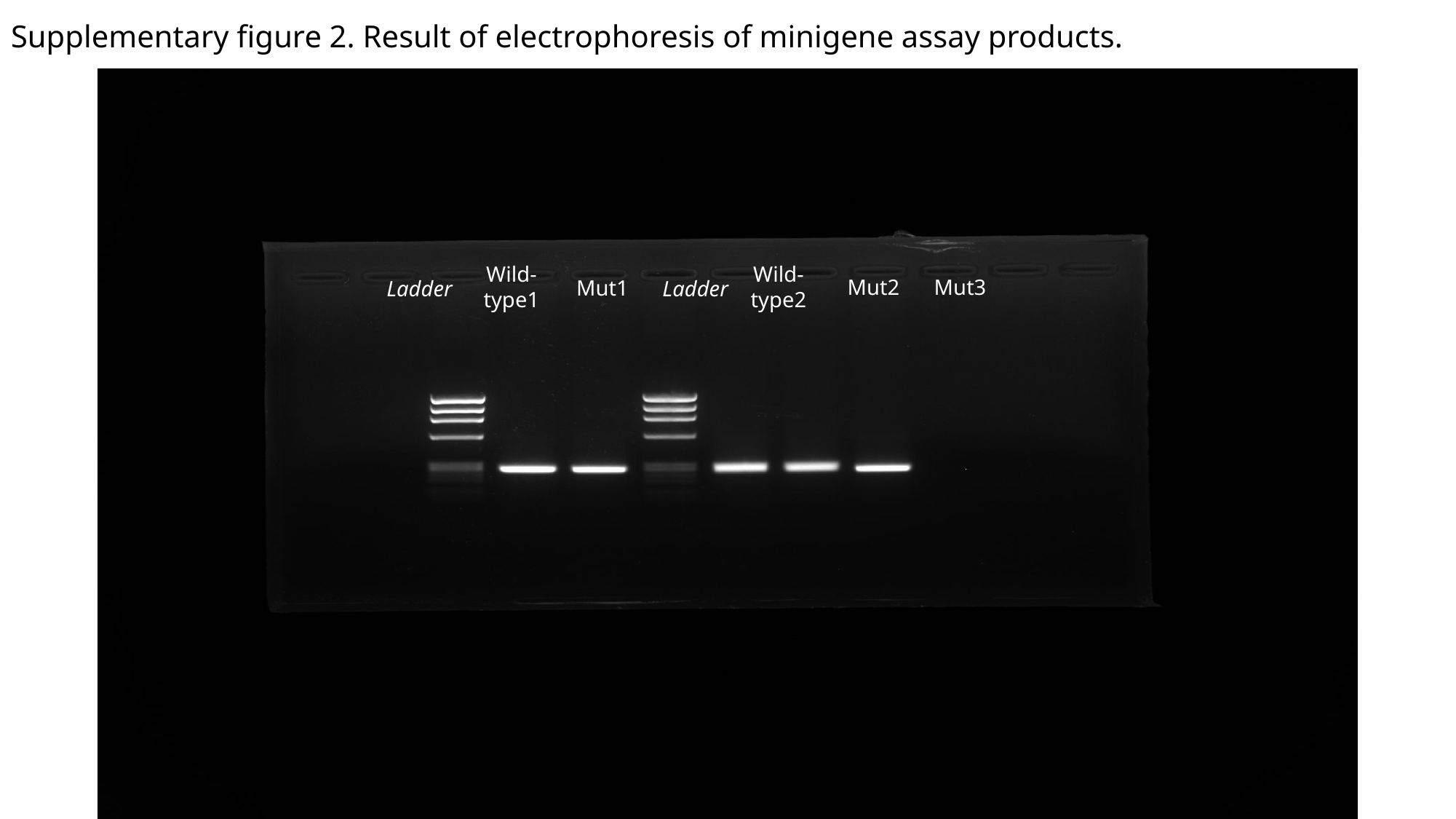

# Supplementary figure 2. Result of electrophoresis of minigene assay products.
Wild-
type1
Wild- type2
Mut2
Mut3
Mut1
Ladder
Ladder

## Slide 3
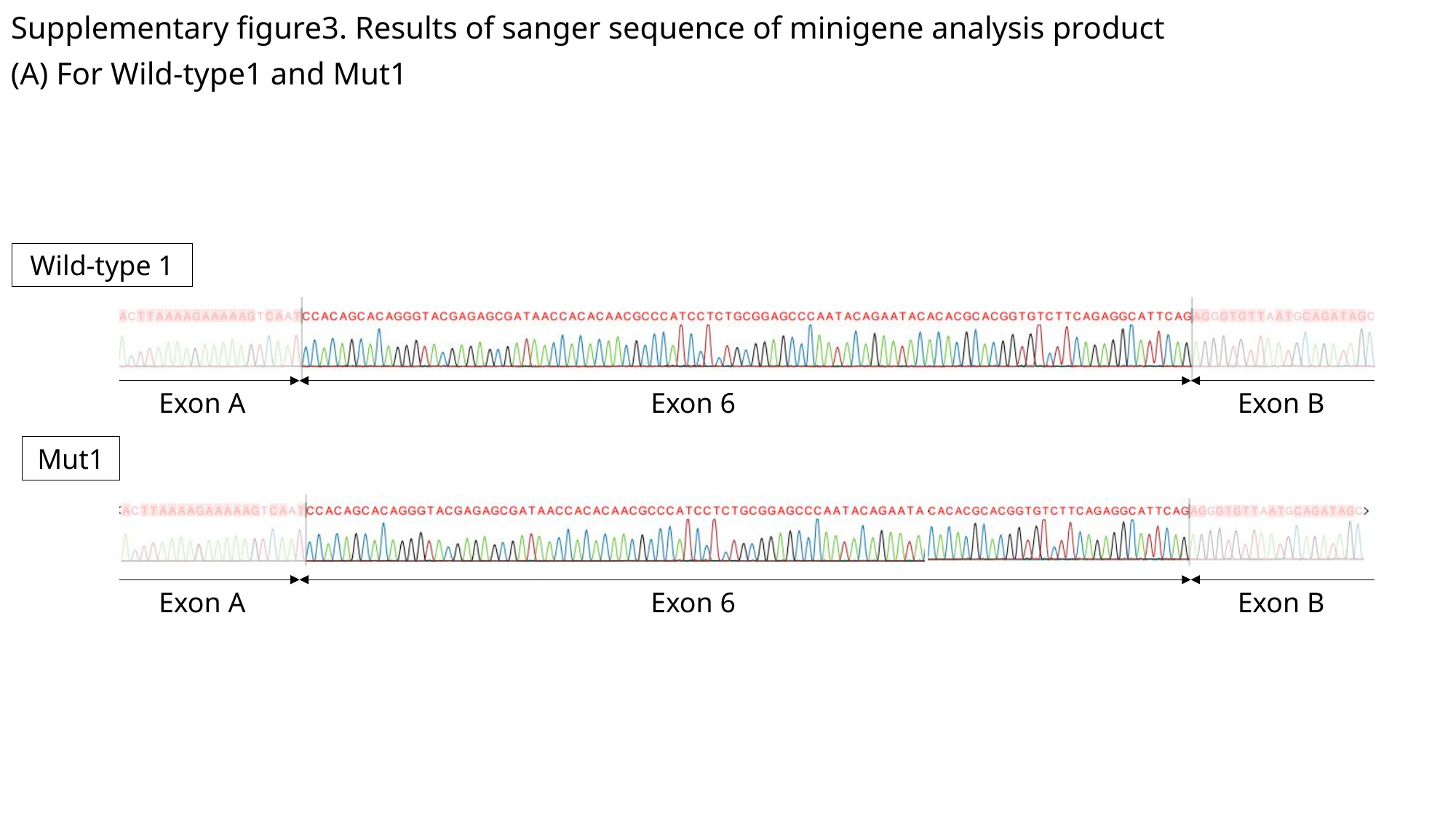

Supplementary figure3. Results of sanger sequence of minigene analysis product
(A) For Wild-type1 and Mut1
Wild-type 1
Exon A
Exon 6
Exon B
Mut1
Exon A
Exon 6
Exon B

## Slide 4
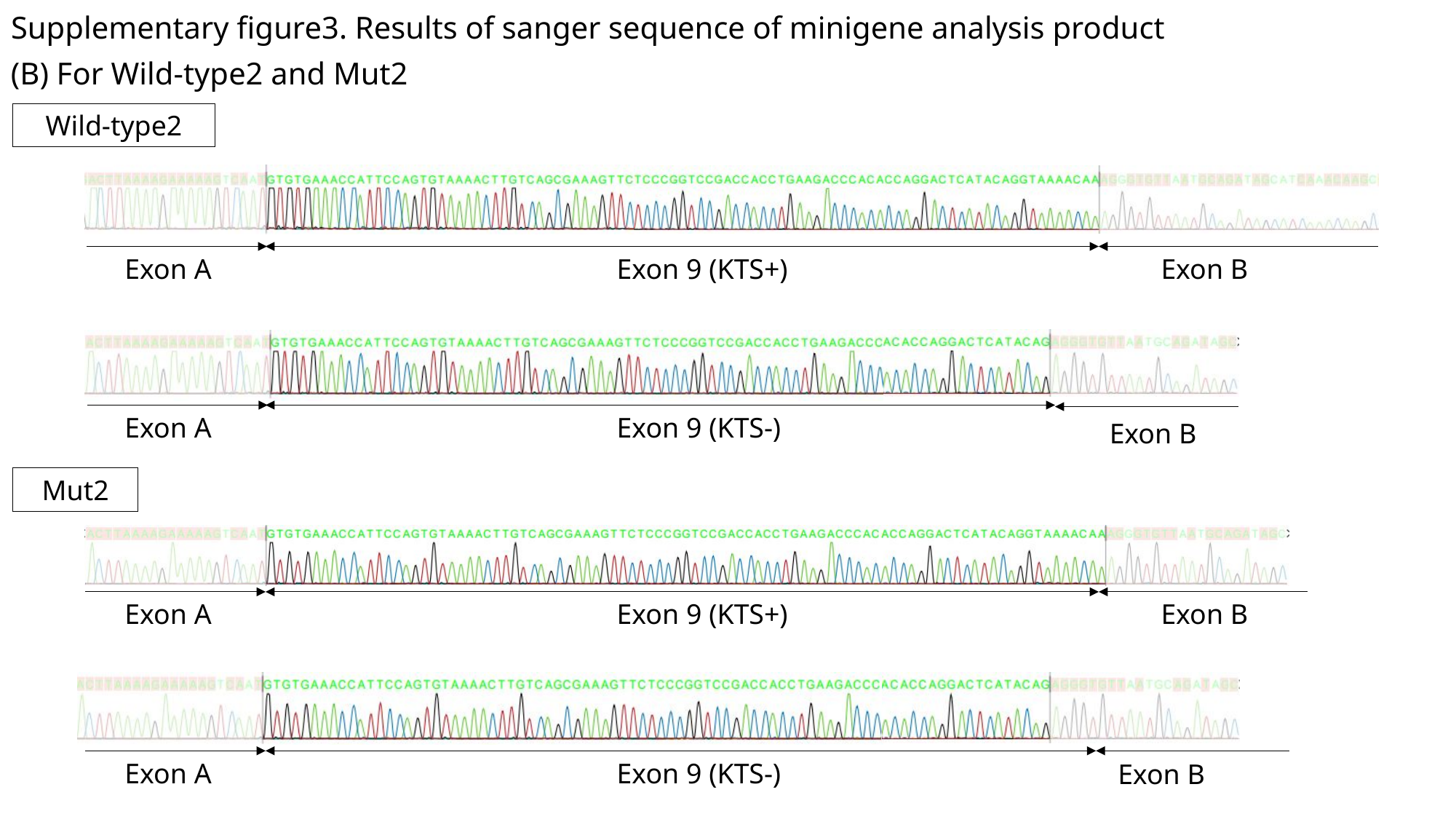

Supplementary figure3. Results of sanger sequence of minigene analysis product
(B) For Wild-type2 and Mut2
Wild-type2
Exon A
Exon 9 (KTS+)
Exon B
Exon A
Exon 9 (KTS-)
Exon B
Mut2
Exon A
Exon 9 (KTS+)
Exon B
Exon A
Exon 9 (KTS-)
Exon B

## Slide 5
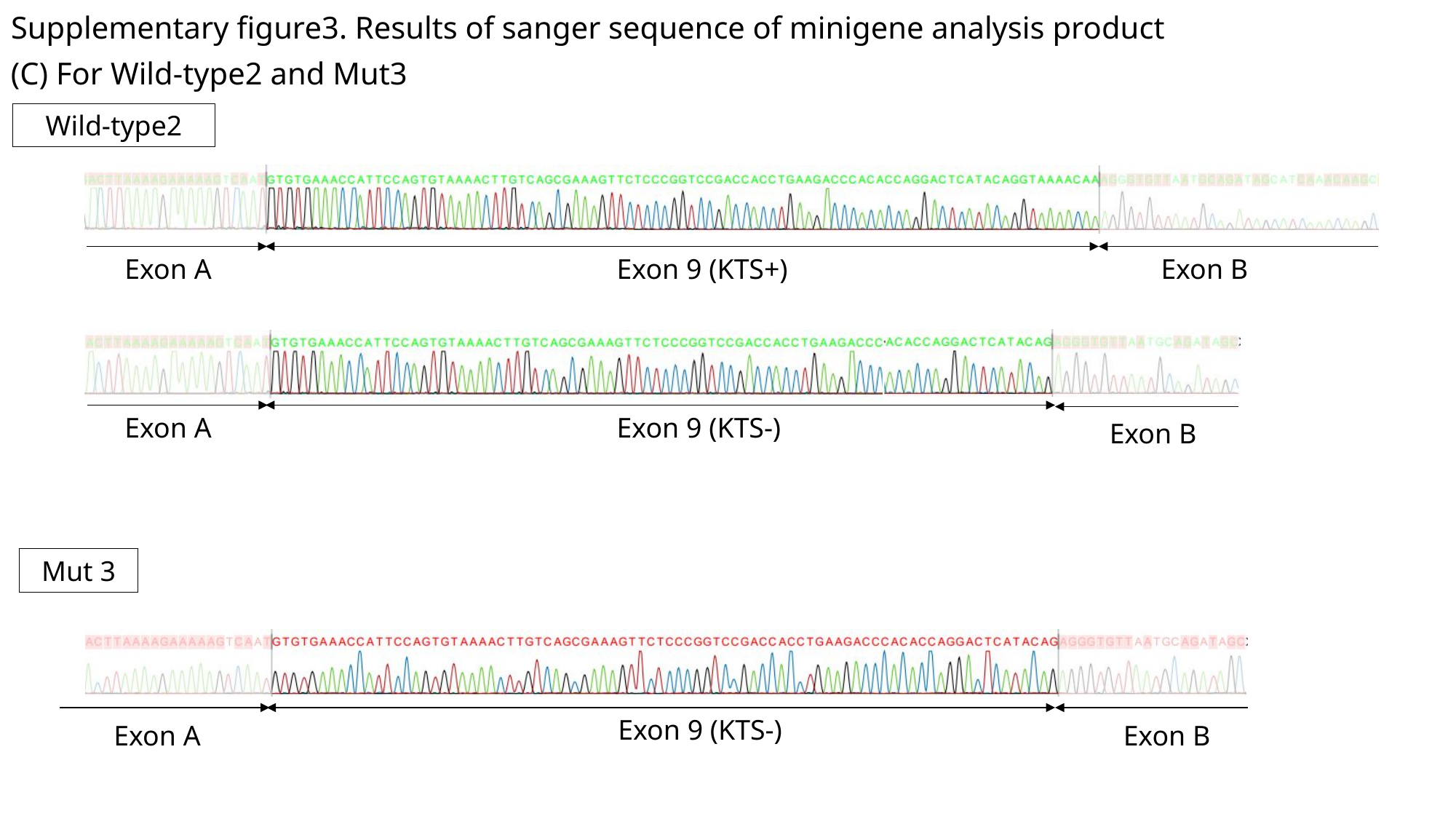

Supplementary figure3. Results of sanger sequence of minigene analysis product
(C) For Wild-type2 and Mut3
Wild-type2
Exon A
Exon 9 (KTS+)
Exon B
Exon A
Exon 9 (KTS-)
Exon B
Mut 3
Exon 9 (KTS-)
Exon A
Exon B
